# Attuning to laboratory animals and telling stories: Learning animal geography research skills from animal technologists

**DOI:** 10.1177/0263775818807720

**Published:** 2018-10-29

**Authors:** Beth Greenhough, Emma Roe

**Affiliations:** School of Geography and the Environment, University of Oxford, UK; School of Geography and Environmental Sciences, University of Southampton, UK

**Keywords:** Animal geographies, animal research, attunement, ethics, ethnography, storytelling

## Abstract

Posthumanism has challenged the social sciences and humanities to rethink anthopocentricism within the cultures and societies they study and to take account of more-than-human agencies and perspectives. This poses key methodological challenges, including a tendency for animal geographies to focus very much on the human side of human–animal relations and to fail to acknowledge animals as embodied, lively, articulate political subjects. In this paper, we draw on recent ethnographic work, observing and participating in the care of research animals and interviewing the animal technologists, to contribute to the understandings of life within the animal house. In so doing, the paper makes three key arguments. Firstly, that studying how animal technologists perform everyday care and make sense of their relationships with animals offers useful insights into the specific skills, expertise and relationships required in order to study human–animal relations. Secondly, that animal technologists are keenly aware of the contested moralities which emerge in animal research environments and can offer an important position from which to understand this. Thirdly, that storytelling (exemplified by the stories told by animal technologists) is a useful resource for animal geographers to engage with complexity in human–animal relations.

## Introduction

Posthumanism has challenged the social sciences and humanities to rethink their anthropocentric approach and take into account the role of more-than-human agencies. Within geography, actor–network theory has played a key role in drawing attention to animal agency (see for example Whatmore and Thorne, 1998), but some argue that it fails to grapple with the more challenging question of how can we study *with* animals their experience of participating in more-than-human worlds (Bastian et al., 2017) and how they might have, and more importantly express, a vested interest in changing and developing these. As Henry Buller (2015, see also Hodgetts and Lorimer, 2014) notes, this poses key methodological challenges, not least the need to overcome a tendency to focus on the human side of human–animal relations and to fail to acknowledge animals as embodied, lively, articulate political subjects. In other words, within Euro-American contexts, there is a common tension between understanding animals-as-a-resource for humans and the opposing position of animals-as-independent of human’s meanings and values. One way in which animal geographers, and those in allied fields such as animal studies and multispecies ethnography (Kirksey and Helmreich, 2010), have sought to respond to these challenges is through learning from others whose work necessitates gaining insights into ‘nonhuman experiences of humanized spaces’ (Hodgetts and Lorimer, 2014: 2). For example, Van Dooren et al. (2016: 1) enrol ‘scientists, farmers, hunters, indigenous peoples, activists, and artists’ in developing their ethological and ethnographic approach, thereby drawing on a host of both Western and non-Western ontologies to try and expand human modes of relating to animal others. Following Buller’s (2015) assertion that animal welfare practitioners provide one example of how to try and see things from the animal’s point of view, we suggest that laboratory animal technologists (ATs), as those responsible for the day-to-day care, welfare and husbandry of laboratory animals, may also have something to teach animal geographers.

Laboratory animal science is a particularly compelling site to explore questions of animal geographies. Indeed animal research in many ways is premised on the ability of humans to attune to the bodies of animal others, to recognize the shared genetics, physiology and vulnerabilities (engineered or otherwise) that allow both diseases and knowledge of them to cross and challenge species lines (see also Kirksey and Helmreich, 2010) and the potentiality (Friese, 2013) of animal bodies to model human ones. Simultaneously, it is also a site shaped by practices of intensive management and regulation and the legacies of scientific disciplines that promote standardization and objectivity which tend to ‘under-value the local, contingent and practical engagements that make health [and we would add welfare for both human and animal] possible’ (Hinchliffe, 2015: 28). Scientific accounts of laboratory animal research pay little attention to how quotidian practices and questions of animal care and husbandry take place (Friese, 2013). In contrast, this is the starting point for our analysis. Firstly then, this paper argues that studying how ATs perform everyday care and make sense of their relationships with animals offers useful insights into the specific skills, expertise and relationships required in order to study human–animal relations.

Our second key argument is that ATs are keenly aware of the contested moralities which emerge when caring for laboratory animals and can offer an important position from which to understand this. In the second of his recent progress reports on Animal Geographies, Henry Buller (2015: 374) suggests there are ‘two central investigative challenges for the social sciences’, and geography’s, recent engagement with the ‘animal’: what can we know of animals, and what might we do with that knowing?’ We argue these questions are shared by laboratory ATs who (as we explore below) on the one hand support the instrumental relation between science and research animals, but who on the other hand also draw on their ability to ‘know’ or become attuned to the animal in their care, enabling them to tell stories about laboratory animal lives which challenge and resist the animal’s reduction to passive objects exploited for purely instrumental ends. Through studying the experiences of ATs, this paper also responds to the emerging agenda within the social science and humanities of laboratory animal lives, and in particular the need to understand how ‘the emotional, embodied and affective relations between animals and people shape animal research and care practices’ (Davies et al., 2016, unpaginated). Our third key argument is, then, that understanding the ways in which those who work with animals use storytelling to negotiate the paradoxes and contested moralities which characterise their day-to-day practice is a useful method for animal geographers. The stories told by those whom work with animals offer insights into the challenges, complexities and contradictions that shape human–animal relations, a means of acknowledging animal agency and a potentially valuable resource for developing politically and ethically engaged interventions and public engagement.

Below we draw on the experiences of the ATs we interviewed and observed, our own ethnographic experiences, and experiences evidenced in the accounts of others working on laboratory animal lives. We argue for (i) the importance of studying ATs; (ii) the ways in which they navigate the paradox at the heart of laboratory animal husbandry; and (iii) the role of the stories they tell in helping perform this navigation in a way that makes the animals in their care differently and more agentively present. We begin though with an introduction to recent social and cultural perspectives on animal research and to our conceptual and methodological approach.

## Animal geography in the laboratory animal house^1^

In focusing on laboratory animal lives we build on a growing body of work concerned with the practice, governance and ethics of animal research. This work has drawn attention to: the historical development of the sites, infrastructures, personnel and models of animal research (Druglitro, 2016; Kirk, 2016); the competing and contested meanings and values (moral, scientific, economic) associated with laboratory animals (Kirk, 2016; Svendsen and Koch, 2013); the ways in which the practice of animal research configures human–animal relations in distinctive ways which demand a reconceptualization of notions of care and ethics (Birke et al., 2007; Davies, 2012; Giraud and Hollin, 2016; Greenhough and Roe, 2011, 2017; Holmberg, 2011); and the implications of recognising animal agency for experimental design and the (co)production of scientific knowledge (Despret, 2004; Friese, 2013).

Like animal geographers more broadly, those seeking to engage with laboratory animal lives have also employed a range of methods, ranging from the more case-study based approaches of philosophers (Despret, 2004; 2013), to archival work charting the social and material construction of laboratory animal infrastructures and personnel (Druglitro, 2016; Kirk, 2016), to ethnographic engagements with the sites, spaces and human and nonhuman agencies engaged in animal research (Birke et al., 2007; Davies, 2012; Friese, 2013; Greenhough and Roe, 2017; Holmberg, 2011; Svendsen and Koch, 2013). What is distinctive about our approach in this paper is that we wish both to offer ethnographically informed insights into how ATs perform everyday care work and negotiate the challenge of paradox of caring for laboratory animals, and also ask what animal geographers might learn from the ways in which ATs use storytelling to narrate and negotiate these practices and challenges.

Between 2013 and 2015 we undertook a two-year research project, funded by the Wellcome Trust, which sought to better understand the key role we felt ATs played in putting ethics into practice (see also Greenhough and Roe 2017). Previous studies have tended to focus more on the scientists and researchers who design and carry out experimental work on animals (see for example Friese, 2013; Svendsen and Koch, 2013). We chose instead to focus on the ATs who carry out the day-to-day labour of laboratory animal husbandry. We asked how their performance of laboratory animal care and monitoring tasks was shaped and conditioned by the ethical review processes and welfare protocols which govern UK animal research, as well as the political and economic constraints of private or public sector research and the experimental requirements of ‘good’ science. Yet what we also noticed through the course of our work was the highly skilled way in which ATs formed relationships with the animals in their care; animals who often had unique needs and requirements as a result of breeding and genetic alteration. The relationship between AT(s) and the animal(s) they work with is one performed through both routine and exceptional care practices. ATs are sensitive to animal suffering, physiological signs and vocalisations, and attentive to how stage of life affects behaviour and to positive or negative signs of health. There is much for animal geographers to learn here to inform research practice in animal worlds beyond the animal house, as well as understanding life within.

Our findings are gathered from our experiences of working as ATs for a week in UK university facilities, as well as the time we spent talking (both formally in interviews and informally on tours of animal houses and at conferences) to laboratory animal technicians and other stakeholders about their work, the skills they seek to develop and their relationship with the other animal house inhabitants (cage-washers, cage-dwellers, escapees, the dying, the newborn, the sick, the research scientists, the postgraduate and undergraduate students, the manager, the salesperson, the instructor, the vet, the NACWO,^2^ the NATCO,^3^ the inspector, the pets). Participant observation can provide detailed insights into day-to-day care and monitoring practices (e.g. adding environmental enrichment, killing), which are hard to capture in an interview conversation, and has proved particularly effective in illuminating human–animal relationships in laboratory animal science (e.g. Birke et al., 2007; Friese, 2013; Holmberg, 2011; Svendsen and Koch, 2013). Repeat interviews with seven junior ATs over the course of two years, discussing their experiences of trying to do the right thing and in some cases going along with them in the course of their day-to-day work, allowed us to explore how ATs’ skills, experiences and responses developed over time and build the rapport and trust necessary to tackle challenging encounters of multispecies relating. While we watched the ATs become attuned to the animals they worked with, and vice versa, the ATs and the animal geographers were also becoming attuned to each other.

Our field sites, largely due to questions of access and the small scale of the study, were UK university facilities with whom we had worked previously or had had previous contact. Perhaps not unsurprisingly this is a sensitive area in which to conduct research, and it takes time to build relations with an animal research community for whom the threat of infiltration by animal rights extremists remains a fairly recent memory. We supplemented these intensive studies with interviews with stakeholders from across the animal research industry, including some pilot interviews at private sector facilities. Our range of species too might, to the un-initiated, seem rather narrow for those seeking to offer a multispecies account. Rats and mice account for 72% of the 2.02 million experimental procedures completed in 2015 (Home Office, 2016: 10), so the majority of human–animal relations we both observed and participated in were between humans and rodents. What such statistics fail to capture, however, is the widespread variability within species, and thus specialized care needs varied between the many different strains of mice bred or engineered to model specific human health or disease states. Our small sample of human subjects was also diverse. While the junior technologists we focused on were all relatively young (estimated to be under 30), they comprised both men and women, and had backgrounds ranging from graduate and postgraduate level education in an animal related area (e.g. marine biology, veterinary nursing) to those with no post-16 qualifications. What they did have in common was a love of and ability to attune to nonhuman animals.

It is only through the process of analysing our findings that we turn to characterize this work specifically as animal geography, where it might otherwise have been framed as a simple study of the work of being an AT. We do so here because while the focus of our study was nominally ATs, what we observed, participated in, talked about and ultimately intervened in was a set of relationships between humans and animals in the laboratory animal house. Central to what we uncovered were a whole host of conflicting multispecies relations between humans and nonhuman animals, ranging from those we expected (between ATs and cages filled with mice) to those we did not, including: discussions of pets, past and current; the presence (and absence) of micro-organisms; the traces of other animal encounters (both material and cognitive) we might have carried with us and might carry away; and the ways in which our work led us to ask, and still leads us to ask, uncomfortable questions about relations with other species across all aspects of our personal and professional lives.^4^ In short, while other styles of doing animal geography exist (see for example Anderson, 1995; Buller, 2004; Lulka, 2008) this fieldwork found us learning from the ATs both how to attune to animals’ lived experiences and how to deal with the moral contestations that arise from attuning to laboratory animal lives.

## Attunement

Hodgetts and Lorimer (2014: 2) suggest that methods involving participant observation of human–nonhuman relations and interviews with human subjects lead ‘to a retention of the bias towards human sensings of non-humans’. Given these constraints practitioners of animal studies, multispecies ethnography and animal geographers have spent time cultivating the arts of ‘attunement’ (Despret, 2004), ‘embodied communication’ (Despret, 2013: Greenhough, 2012; Haraway, 2008), ‘noticing’ (Tsing, 2015), ‘attentiveness’ (Van Dooren et al., 2016; Druglitro, 2016), ‘impression’ (Hayward, 2010), ‘symphysis’ (Acampora, 2006; Holmberg, 2011), ‘response-ability' (Greenhough and Roe, 2010; Haraway, 2008) or ‘learning to be affected’ (Lorimer, 2008) by nonhuman others. While not entirely commensurate, each of these wide-ranging terms gestures towards a need to pay attention to the fleshy bodily and emotional susceptibilities, potentialities and vulnerabilities of nonhuman others, engaging with how we learn from another (and they learn from us) through multi-sensual embodied encounters, along with cognitive reflection. In the discussion that follows, we adopt Despret’s (2004) term ‘attunement’ as one specifically developed to capture relations between research animals and those who work with them. Attunement aligns us with broader moves in animal geography towards ‘some emergent knowing of non-humans: their meaning (both materially and semiotically); their “impact” on, or even co-production of, our own practices and spaces; and our practical and ethical interaction with and/or relationship to them’ (Buller, 2015: 379). In this section, we seek to define and nuance a particular understanding of attunement as practiced by ATs, drawing attention to the specific skills, expertise and relationships they have developed towards the animals in their care, and to reflect on the ways in which AT-animal relations and laboratory animal house environments both constrain and enable different kinds of attunement.

### Seeing mice skilfully

There is a hotbox (incubator) for mice recovering from surgery. It contains some mice with bandages from skin grafts. On the side is another tool, a chart of pictures illustrating the Mouse Grimace Scale – an illustrated list of facial signs of stress and discomfort. We discuss how difficult it is to see the differences between the pictures, and Debbie and Fiona^5^ describe it as a knack, one you develop. I look at the series of animal faces but struggle to see the differences between them. (Fieldwork diary, July 2014)

The Mouse Grimace Scale (MGS) was developed in 2010 (Langford et al., 2010) as a means of coding (seemingly objectively) pain in mice based on changes in facial expression, having previously been developed for other mammalian species. Using the MGS depends on the ability of ATs to, in Druglitro’s (2017: 10) terms, ‘see skilfully’, supplementing the objective ‘informed gaze’ they have gained through, for example professional training courses in animal behaviour, handling and physiology, with more tacit knowledge developed through experiences of handling and being with animals (Birke et al., 2007; Holmberg, 2011) and through the shared sensitivities developed by experiences of being bodies together, in proximity (Acampora, 2006; Despret, 2004; Greenhough and Roe, 2011). The MGS is not without its critics (see Doughman, 2015 who feels it can mislead in individual cases, if not the population as a whole). Animal geographers have similarly cautioned an over-reliance on visual senses as means of knowing animal worlds in light of the taxonomic partialities such sensings may induce (Lorimer, 2006b). It is relatively straightforward to design grimace scales for animals that are big-like-us and mammals-like-us, less so to design scales which register expressions of pain and discomfort in frogs or fish. From our observations ATs had developed a skill of reading murine bodies as well as faces (through the MGS) that offered them a way to access murine experiences. It takes a particular kind of skill to read suffering in mice based on changes in facial expression, one we (as ethnographers) struggled to acquire. Drawing on Despret (2004), we would suggest the process of ‘seeing skilfully’ is an act of ‘anthropo-zoo-genetic practice’ whereby animals and humans attune to each other through the ways in which humans manipulated, handled, caressed, fed and encouraged the animals they care-work with.

Furthermore, ATs not only learn sensibilities to different murine facial expressions, but also to sense differences between the various strains of mice who inhabit animal houses. There are hundreds of outbred, inbred and transgenic strains of mice whose bodies have been particularly tailored to model forms of disease or whose bodies have been made to suit scientific requirements – for example to have no gut microbiome. The Jackson Laboratory, one of the main suppliers of inbred mouse models, maintains over 75 different strains. Distinguishing between them requires a different kind of gaze:

In the zoo you take a considered route amongst different species, but here it is strains or genotypes or different researcher’s mice that map the layout of the room. My species-centric zoo-gaze doesn’t know what to go on to look for, how to recognise difference in the mice. There is different information offered through sticker colours, card colours, birth dates, codes etc – but I fail to make any of the mice a different type of exhibit. (Fieldwork diary, March 2015)

Consequently, in the laboratory animal house we explore not only multispecies relations but multistrain relations, because, ‘every strain of mouse is different [...] they’re genetically modified mice so they’ve all got their own little characters’ (Interview with Claire, Junior Animal Technologist, November 2013). Gradually, from spending time with different strains, differences start to emerge. Some of these are visual, such as the black colouring which marks out C57BL/6, one of the most commonly used strains, at other times differences are more subtle and take handling experience: ‘sometimes you can detect the difference between a wild type and a genetic one just by picking it up and the behaviour it does’ (Interview with Claire, Junior Animal Technologist, November 2013). Importantly then, while the MGS offers a guide to reading murine faces, it is ATs who attune to the facial and other bodily expressions as characteristics of the strains in their care.

### Care

Jack sometimes surprises me. Just as I have decided he's fairly instrumental about his work, he'll do something that shows he thinks about the animals and their experiences. For example, today he adds a red plastic hut to a breeding cage containing one male and two females, one of whom is pregnant. He explains that as there is another female in the cage, this will give her (the pregnant female) some privacy, ‘giving her a bit of space for herself’. (Fieldwork notes, September 2013)

Within the laboratory animal house, attunement is a resource drawn on both instrumentally (as part of fulfilling requirements to monitor and promote good animal welfare) and affectively (as a resource to develop the capacity for animal care; seen as central to being a good AT (Greenhough and Roe, 2017)). Reminiscent of Despret’s (2004, see also Candea, 2013) arguments for researchers to be better attuned to the animals they work with and to use this to inform research design, advocates of laboratory animal welfare have long insisted that the kinds of care practiced by ATs who are attuned to the needs of their animals is conducive to the production of better (less stressed, healthier) animal models (see also Kirk, 2016 for a historical perspective on this). Consequently, while some suggest commitments to scientific objectivity can ‘sideline the importance of care’ (Davies, 2012: 629), others are concerned with the ways in which some forms of care become co-opted into the experimental system: ‘In certain contexts care is precisely what enables the instrumentalization of life, in being used to gain knowledge about entities that can be exploited for the purpose of control’ (Giraud and Hollin, 2016: 31). Or suggest these practices are focused on conditioning animal bodies in order to stimulate their ‘“potential” as organisms and materials that scientists could use for prescribed purposes’ (Druglitro, 2016: 159, see also Friese, 2013). As Puig de la Bellacasa notes (2012: 198) in an instrumental setting ‘caring is more than an affective-ethical state: it involves material engagement in labours to sustain interdependent worlds, labours that are often associated with exploitation and domination’. Trying to understand how ATs negotiate this paradox is the second key contribution we wish to make through this paper.

Some argue that acts of care, like adding a plastic hut to a cage, both evidence a process of attuning to the needs of animals in their care (Druglitro, 2016) and the presence of an affective resource which resists and or exceeds the more instrumental meanings and values seen to dominate human–animal relations in animal research (Giraud and Hollin, 2016). This non-instrumental form of care is evident in ATs’ innovations of forms of environmental enrichment tailored to the specific species, strain, and/or life-stage of animal in their care (see Greenhough and Roe, 2017), such as the delivery of post-surgery analgesics for mice in strawberry flavoured jelly. What Holmberg (2011: 158) terms ‘the small, practical measures that make an animal’s life and death a bit richer’.

### Contested moralities

There are, however, risks associated with focusing on the role of attunement in shaping multispecies or ‘multistrain’ relations, in that the more attuned we become to intimacies of human and animal bodies learning to live together, the more we perhaps lose sight of structural constraints. Contemporary laboratory animal houses, even within the university sector which we studied, are increasingly framed as service providers. Scientists and researchers are referred to as ‘customers’, for whom laboratory animal care staff provide a service, animal care, and a product, animal models, for which they are then charged through university accounting systems. This economic, technoscientific infrastructure has many implications for the ways in which laboratory animals’ lives, labour and the labour of those who care for them are standardised, prepared, and trained (Druglitro, 2016; see also Kirk, 2016). Situations can be read in multiple ways. For example, group housing is both a means of improving animal welfare (providing recommended limits are not exceeded), but also a means of reducing costs: ‘every week it’s £10 per cage so … like, if you’ve got a cage of three females and two females so they could be put together so that’s £10 rather than £20 they would pay’ (Interview with Eleanor, Junior AT, August 2013).

Particularly significant for this paper and its focus on processes of attunement is the implications this has for the practice of animal experimentation. As animal facilities are increasingly commodified and centralized a separation emerges between those who care for, and are arguably most attuned to, laboratory animals, and those who carry out experiments with them. Such moves effectively redistribute and separate out the ATs who largely bear the emotional costs of animal research (through their attunement to the animals they work with) and the economic ones (borne by researchers who are charged for the care services ATs provide), creating what we might term an ‘emotional division of labour’. At times these trade-offs were stark. For example, many ATs find the process of culling animals emotionally challenging, and prefer to do it on particular days or particular times – when they feel ‘up to it’ – as long as to do so would not lead to an animal suffering. However, researchers who are charged for each animal they have in stock may place pressure on ATs to cull quickly: ‘so because they’re getting charges for them, they’ll be like, “oh, [why] can you not do it now? [...] I don’t want to be charged anymore”’ (Interview with Debbie, Junior AT, November 2013). We therefore share with Geiger and Hovorka (2015) a methodological and theoretical commitment to paying attention to how animal and human lives are co-constituted within uneven cultural economic networks of power and capital that frame the nature of daily encounters.

What emerges is a contested moral economy (Svendsen and Koch, 2013) whereby the affective and emotional values that emerge when scientists, researchers, ATs attune to laboratory animal lives ‘cannot be easily separated from factors that would be properly associated with a political economy of animal-dependent experimental science’ (as noted above), nor from seemingly incompatible scientific processes of rationalization, standardization and objectivity (Kirk, 2016: 167). These are challenging contexts in which to try and advance the interests of animals, contexts where it may be presumed that while we might acknowledge shared bodily vulnerabilities, animals’ needs and animal rights, these are always subsumed to those of humans (see Johnston, 2015). How might research methods for animal geographers develop to better acknowledge the contested moral economy around animal care and use? How, too might animal geographers learn to recognize the instrumental and structural constraints that shape their attempts to attune to animals lives? If we learn anything from the ATs here, it is that the infrastructure imposes an inability to escape animal exploitation, and AT attunement to animals is part and parcel of that. Some theorists strike a more pragmatic, conciliatory tone to these underlying tensions (see Haraway, 2008; Puig de la Bellacasa, 2012; Van Dooren, 2014), acknowledging suffering, harm and death of nonhumans as a consequence of more-than-human living and that humans cannot escape these lively and deathly entanglings. Others take a more empirical approach. For example, Geiger and Horkova (2015) combine research methods from social science (interviews, participant observation, textual analysis) and animal welfare science (animal welfare assessments) to examine the co-constitution of smallholder and donkey lives in low income households in Botswana, concluding that their shared political and economic marginalization has significant (often negative) implications for both donkey welfare and human livelihoods. Neither approach, however, offers a means by which such human–animal relations might be renegotiated or changed. Here therefore we propose that we might take inspiration from the ways in which ATs try to negotiate these tensions by telling stories about their relationships with particular individual animals they have cared for. We argue that these stories not only offer insight into the contested moral economies and structural constraints which shape AT–animal relations in the laboratory, but also that they offer a strategy and a vehicle through which such relations might begin to be renegotiated.

## Telling laboratory animal stories

Lots of people have stories about one special animal they’ve worked with as well, […]. You talk to, particularly some of the older technicians, they’ll tell you a story about one particular animal and that kind of becomes sort of their way of understanding why they’re doing things like this, it’s interesting. (Interview with Claire, Junior AT, August 2014)

In making an argument for the importance of ethnography, Annemarie Mol (2008) entreats us to ‘tell each other stories’. Her plea resonates across work in the social sciences concerned with accessing and understanding relations, including those between species. Such an act speaks not only to the process of becoming (however partially) attuned to the lifeworlds of nonhuman others, but what we do with those sensitivities. As Puig de la Bellacasa (2012: 207) notes, with a closeness of relations comes an accountability, an acknowledgment that ‘creating knowledge has consequences, that those we study are not there only to think-with but also to “live with”’. Once impelled to respond, what form might that response take?

Storytelling refigures relations, multiplying perspectives and capturing forms and moments of encounter which are resistant to more conventional, isolationist and calculative modes of academic writing (Lorimer, 2006a; Puig de la Bellacasa, 2012). Storytelling is also distinctive from the thick description that characterizes ethnographic accounts. Ethnographic writing is evocative and affective, it ‘takes us there’ (after Lorimer, 2006a), it sets out the nature of relations. Storytelling, by contrast, is reflective and thought-provoking (Van Dooren, 2014). Storytelling asks us why we are there and where we might be otherwise. It is both ‘descriptive (it inscribes) and speculative (it connects). It builds relation and community, that is: possibility’ (Puig de la Bellacasa, 2012: 203). There are of course other stories told about laboratory animals. Some of these are designed to act as cautionary tales, like pigoons, the human–swine hybrids that lurk threateningly in the undergrowth of Margret Atwood’s (2009) post-apocalyptic *Oryx and Crake*. Others however, have more in common with the stories told by ATs, which we explore here for two key reasons: Firstly, because they serve to give laboratory animals a particular identity and often a name as seen in Richard Adam’s (1977) *The Plague Dogs*, which follows the tale of two dogs – Snitter and Rowf – escaped from a fictional testing facility. Secondly, as exemplified by Karen Joy Fowler’s (2014) *We are all completely beside ourselves*, they are able to capture some of the ethical anxiety and constant questioning of human–laboratory animal relations that characterises AT’s discourses. Unlike these tales, however, the stories ATs tell are more fact than fiction.

### Storying individuals

Dangerous thing, a name. Someone might catch hold of you by it, mightn't they? (Adams, 1977)

Animal geography faces challenges between focusing on collectives and focussing on individual animals (Bear, 2011; Buller and Roe, 2018; Hodgetts and Lorimer, 2014). On the one hand, a focus on individual animals can be accused of misrepresenting the realities of animal lives, given many animals are rarely treated as individuals but as flocks of chickens, herds of pigs or tanks of exotic pet fish. On the other hand, a focus on collectives leads to accusations of suppressing the ethical imperative of a singular sentient being’s experience. By contrast, within the animal house ‘multistrain’ relations, rather than multispecies relations, necessitates laboratory ATs focus on the individual. Each animal gets an individual examination by sight and/or through handling. Evidently, some animals receive even greater individual attention from ATs than others. ATs often spoke of their care for and relationships with laboratory animals by telling the story of one particularly charismatic individual. Every AT seemed to have ‘a story in their mind’, one which puts a face and a name to the otherwise anonymous figure of the laboratory animal:

I called him Fat Frank because he was just the biggest mouse I’ve ever seen. And I would weigh him just to see how much weight he’d put on. He was just a real fatty. I just, he was a little favourite, he’s gone now ‘cause he was quite an old mouse, but yeah I had one [laughing]. (Interview with Claire, Junior AT, November 2013)

We suggest these stories are interesting because in the space of the interview, or perhaps the space made by the interview, they do a particular kind of work. They reassert the importance of particular encounters – attunements – between ATs and the animals they work for. Lorimer (2007: 921) might describe such encounters as interspecies epiphanies, ‘encounters with a particular organism, or group of organisms, in which they were strongly moved’ and which ‘inscribed a memory and the foundations for a lifetime attachment, interest, and concern’. Furthermore, these stories are invoked in particular ways, most often as a mode of countering those moments of detachment and instrumental forms of value which, as noted above, remain in tension with other modes of engagement between ATs and the animals they work with. These stories acted almost as a kind of ethical refrain, where more instrumental views of laboratory animal life are set against stories of human–animal affection:

I had a girl, Pauline, who worked for me years ago … and she had a pet rat and when she used to clean it out, this pet rat used to sit on her shoulder like a parrot. And I walked past her one day and I said, ‘What’s that rat doing?’ She goes, ‘That’s my pet rat. He sits on my shoulder’. And he did. (Interview with John, Facility Manager, July 2013)

Having ‘pets’ – animals that were given treats, extra attention, cuddles or similar – was a key coping strategy for ATs, allowing them – however temporally – to relate to and engage with the animals in their care in a different way (see also Holmberg, 2011). The pet rat in the laboratory forces a re-thinking of those relations, in a similar manner to Erica Fudge’s (2011) tale of a house mouse with whom she became ‘pest friends’. Gillespie (2016: 577) similarly argues that ‘telling the stories of those whose lives often go unremarked is an act of resistance that makes a statement about whose lives matter and have meaning’. We would argue this practice of storying and naming individual animals is not necessarily about turning animals into ‘furry people’ – (ATs by virtue of their training in ethology and animal husbandry are often acutely aware of differences between species as well as strains) - but about acknowledging their subjectivity, individuality and unique experiences of the worlds which we cohabit, in the manner of Chris Bear’s (2011) experiences of getting to know the octopus, Angelica.

As noted above, in their analysis of a beagle testing facility at UC Davis in the US, Giraud and Hollin (2016) argue that the affective relationships or attunements between laboratory animals and those who work with them are all to readily co-opted to experimental ends. While we agree there is evidence of scientists learning from and actively manipulating the affective qualities of experimental organisms in order to render them more ‘content’ or ‘docile’, the stories told by ATs seem to be doing another kind of work. Not least because they often focus on animals who stand out precisely because they refuse to be docile:

And one of my mates came in [to the animal house] at the weekend, and I saw him on the Monday, and he was cursing me, going, ‘Oh, God, your bleeding cats get on my nerves’. And I said, ‘Why? They’re not my cats, you know, what’s wrong with it?’ He said, ‘He [the cat] jumped down off the shelves’, and he [the mate] was carrying his [the cat's] tray of milk, and it went over, and he [the mate] said, ‘The tray of milk went all over the floor’. (Interview with John, Facility Manager, July 2013)

Telling stories about particular encounters with individual animals acknowledges the agency of an animal other – their capacity to affect us – and how this may motivate us to respond in ways which escape momentarily the moral economy of the laboratory.

### Storying ethicopolitical relations

Puig de la Bellacasa (2011: 89) argues that attunement is an ethicopolitical act in that it generates a sense of obligation to take the needs and experiences of an(nonhuman)other into account, to ‘recruit people into finding better ways of living with [human and nonhuman] others’ (Bennett, 2010: 27) and ‘cultivate worlds of mutual flourishing’ (Van Dooren et al., 2016: 17). For example, Jamie Lorimer (2008) describes how conservationists, through spending long periods of time with corncrakes, are better attuned to the needs of their ‘target organisms’ and thereby both better equipped and motivated to advocate for their cause. More critical approaches (Giraud and Hollin, 2016) suggest that within a laboratory environment such possibilities may be highly constrained, but this in some ways makes the stories told by ATs that much more compelling – here are stories about care which point to the ways in which ‘laboratory humans cannot be content with doing ethics in a calculating, instrumental way’ (Holmberg, 2011; see also Greenhough and Roe, 2011). As one AT put it, in the field of animal technology storytelling becomes a way of trying to balance a hope or conviction that the research their labour is supporting will have significant benefits for both human and animal health and the specific costs to individual animals they witness:

You remember the faces and names of some of the animals you have worked with … Everyone has a story in their mind. It's important to be able to ‘attach that monkey's face to this goal’. (Extract from interview notes with Chris, Junior AT working at large Contract Research Organisation, November 2012)

We suggest Haraway’s (1997: 267) concern with storytelling may be linked back to her interest in the role of the ethnographer as a modest-witness: ‘Witnessing is seeing: attesting, standing publically accountable for and psychically vulnerable to one’s visions and representations … fraught with the consequences of unconscious and disowned desires and fears’. This vision of the figure of the witness is distinctive from readings that may, for example, see the role of ‘witness’ as closely linked to regulation, a responsibility of oversight bodies such as the Animal Experimentation Council (or in a UK context the Home Office) who provide scrutiny to ensure animal research is conducted in accordance with accepted protocols and norms (Druglitro, 2017: 16). It is also distinctive from readings that suggest the role of bearing witness might find its animal research analogy in the figures of anti-vivisectionist protest, for whom ‘the act of witnessing animals’ predicaments, and then sharing their stories, is a political act that resists the erasure of individual animal lives, suffering, and deaths’ (Gillespie, 2016: 575). What is striking in Gillespie’s account of witnessing the lives (and suffering) of dairy cattle is that in empathising with the cattle she seems to end up presenting the humans in her story in a caricatured way. Their inability to relate to the animals in their care is held up for critique as they engage in ‘jovial’ and ‘light-hearted banter’. We cannot ignore the humans in this multispecies story any more than we can ignore the animals:

I think seeing facilities and not engaging with those who work there at the same time, is problematic… I remember the tears when we spoke with someone who worked with the [name of facility] primates. It's about putting the animal lives in context, rather than making it turn into a zoo trip… (Extract from online research team discussion, 2017).

What the stories told by ATs might do, therefore, is bear witness to both the human and animal costs of animal research.

Telling stories is one way in which to make public the human anxieties that surround animal work. The world outside the laboratory animal house can react dispassionately towards those humans working in these spaces, whilst showing compassion to the animals. For ATs, ours and others research interventions into their workspace and the stories we tell are important, as it can allow other understandings of laboratory space to be shared and transmitted, challenging conventional assumptions about laboratory animal lives:

Well you feel a bit vulnerable in public places, like I wouldn’t talk about it in a public place for that fear of someone’s adverse reaction. And you’ve seen things on the news about how they dug up graves of people, didn’t they…and that is just so extreme. It does make you a little bit wary. But I think we should talk about it more and then people will learn that it’s not as bad as they think. (Group AT Interview, September 2013)

There is a curious role reversal in this quote with the usually marginalized figure of the antivivisectionist protesting voice now strangely threatening and empowered, a voice which, while opening some things up to critique, also closes others down. This makes us reluctant to further deaden the impact of these animal stories by presenting them as only one mode of relating among many. As Haraway cautions,

It matters what matters we use to think other matters with; it matters what stories we tell to tell other stories with; it matters what knots knot knots, what thoughts think thoughts, what descriptions describe descriptions, what ties tie ties. It matters what worlds make worlds, what worlds make stories. (Haraway, 2016: 12)

A classic STS move would be to tell many stories, stories which multiply relations between ATs and experimental animals in the lab (cf. Svendsen and Koch, 2013: S127) or conservationists and species in the wild (cf. Candea, 2013). In the same vein, we might set against the tales of ‘Fat Frank’, and other individual animals for whom ATs developed a particular affection, their relationships with other less engaging, charismatic or even unappealing individuals. Or we might stress the ways in which the sheer economics of scale in the mouse room, which may be home to hundreds of mice at any one time, belie the kinds of close affectionate relationships described above. We might also talk about how the repetitive nature of husbandry tasks – cage cleaning, checking plugs to see if mice are pregnant, weaning, culling – provides scope for tuning out as well as attuning to nonhuman others (something we even found ourselves doing in the course of a week long fieldwork engagement). Or how the affective presence of nonhuman others gets weighed against other scientific and economic forms of value as part of a contested moral economy. Somehow though, to tell other kinds of stories here would be to dismiss the importance of the particular stories we share above; the work they can do, the work the ATs we spoke them arguably *want* them to do. This is evident in the way ATs themselves allude to the multiplicity of meanings and relations sketched out above:

Although you shouldn't get attached, I do love the ones that will just sit and you can sort of–, or the ones you make better you sort of just think I’ve helped a little bit, you know what I mean. I enjoy working with animals and I sort of feel good that, although animal testing and everybody thinks that’s bad, I just think well I’m not doing it because I–, well, it’s not that I don’t agree with animal testing, I think it’s sort of needed. But I feel like I’m helping the animals that have to be tested and to sort of fix things. So I feel sort of good about that, and that’s the bit I enjoy. I enjoy working with animals and sort of making sure they’re okay. (Interview with Eleanor, Junior AT, August 2013)

In Eleanor’s musings on the attachments (attunements) she forms with the animals in her care she does not refuse the more instrumental sense that ‘the animals have to be tested’, but the stories she wants to tell about her relations to those animals, about working with them, helping them, making sure they are OK. So here, we make a deliberate decision to foreground particular laboratory animal stories as told by laboratory ATs because they matter. In this way, we are following in the *Flightways* of Van Dooren (2014), whose beautifully told stories of multispecies entanglements serve to sensitise us to particular human–nonhuman becomings of which we were largely (with the exception of the scientists, ethologists and wildlife workers Van Dooren travels alongside) previously unaware. Unlike Van Dooren, however, we choose to foreground the stories told to us, rather than our own accounts. These stories are important, we argue, because they matter to the ATs who share them. They matter for the ways they affectively resonate with the unsettling nature of animal technology work, of the need to constantly resist and push up against the more objective and instrumental relations and values which operate within laboratory environments. Beth can still recall the shocked and questioning silence that filled the room the first time she asserted that ‘animal labs are full of animal lovers’. These stories matter for the way in which they memorialise particular, non-instrumental ways of valuing laboratory animal lives. They matter because they are stories not often told and arguably even less often properly listened to. Our third key argument therefore is for animal geographers to pay attention to the stories told by those working with animals in order to better understand the ways in which they negotiate complex human–animal relations and the wider social and political-economic infrastructures within which they are embedded.

## Conclusions

Compared to the ATs we observed and momentarily worked alongside, our entanglements with laboratory animal lives were more fleeting. Our entanglements were also differently invested those shaped by the instrumental goals of research using animals which frame animal bodies as a resource. So whilst, in our role as multispecies ethnographers working towards being multistrain ethnographers, we may observe the methods and even adopt the practices of laboratory ATs, our skills and experience are necessarily different and arguably more limited. Nonetheless, we suggest here that the affective relations and processes of attunement seen in the spaces of animal research might make three key contributions to the study and practice of animal geographies.

Firstly, studying animals requires specific skills, expertise and relationships, evidenced in the ways in which ATs use the MGS as a device for learning how to attune to the lived experiences of the mice in their care. Animal geographers who have limited experience of working alongside, for example, specific strains of mice, will have limited ability to be reliable witnesses of murine suffering, but we can learn to recognise both the skills those who work with animals develop in atttunement and how this then shapes the ways in which they relate to those animals in their care. Such attunements suggest animal geographers need to not only advocate for the recognition of animal agency within the academy, but to understand how such recognition happens in a wide range of (sometimes unexpected) sites, spaces and contexts, sometimes intuitively, often through training or experience. Furthermore, studying how ATs attune to laboratory mice draws our attention to the specificities of human–animal relations, which (in the case of animal research) comprise not only multispecies but multistrain relations. We therefore argue that animal geographers need to explore how humans attune not only to differences between species, but differences between breeds and strains.

Secondly, paying attention to the specific skills, expertise and relationships ATs have developed towards the animals in their care also allows us to reflect on the ways in which AT–animal relations and laboratory animal house environments both constrain and enable different kinds of attunement. At the heart of the role of ATs is the tension between the need to provide care and how this is harnessed by laboratory animal science for utilitarian ends through the endeavour of producing compliant and useful bodies for science. In studying paradoxical human–animal relations where care and attunement become instrumentalised, and in attending to the moral anxiety expressed by animal care takers around their work, we contend animal geographers might find a generative space for enquiry. Furthermore, ethnographic experience within the commercial infrastructures of animal research reveals how these relations and spaces impose limitations not only on animals, but on those who care for them, and indeed those who research such sites and spaces. Close ethnographic scrutiny also offers insights into hidden costs (such as the emotional labour born by ATs) and benefits (the pleasure of working closely with nonhuman others) of both animal technology and animal geography.^6^ This is a lesson which is particularly foregrounded by the animal research context, but one which perhaps also could be more thoughtfully and reflexively engaged in other animal geography work (farming, pet-breeding and keeping). We argue that moral anxiety might therefore be embraced as a condition as well as a key object of animal geographers’ research.

Thirdly, animal geographers need to pay attention to the stories told by those working with animals in order to better understand the ways in which humans negotiate complex and contested human–animal relations and the wider social and political-economic infrastructures within which they are embedded. ATs story animal lives in ways that bear witness not only to animal suffering, but to animal agency, rendering laboratory animals both lively and grieve-able and hanging on to the speculative desire, if not the immediate possibility, of seeking other ways of living together. Telling stories allows ATs to avoid becoming desensitised to animal lives, staying with the troubling paradox of caring for laboratory animals and refusing to see either themselves or their animals as docile bodies. We find within these narratives a recovery of animal and AT agency and subjecthood, revealing more complex, contradictory and care-full human–nonhuman relations in the laboratory. While storytelling has featured in animal geographies as a methodological approach (Lorimer, 2006a) and mode of writing (Van Dooren, 2014) we argue much greater use could be made of the stories narrated by research participants as a means of evidencing the complex and ongoing negotiation of human–animal relations, and as a means of balancing out the often politically flattening nature of work that focuses on multiplying and cataloguing different sites and modes of human–animal encounter. While previous work has drawn attention to the conceptual significance of acknowledging the suffering, harm and death of nonhumans as a consequence of more-than-human living (see Haraway, 2008; Puig de la Bellacasa, 2012; Van Dooren, 2014), and to the significant impacts of political-economic structures on the lives and livelihoods of marginalised animals and humans (Geiger and Horkova, 2015), here we argue for attention to the ways in which human–animal relations are storied by those engaged in them. By sharing these laboratory animal stories, as told by those who live and work alongside them and are most attuned to their welfare, we hope to offer a vehicle through which such relations might begin to be reconceptualised and renegotiated. Our future work will build on the promise of AT stories, drawing on these as a resource to create public engagement platforms for staging (we hope) more nuanced, politically and ethically engaged debates about vivisection which better acknowledge the complexities of the animal research environment.

## Notes

1 We use the phrase ‘animal house’ here as a colloquialism for what are more usually termed Biological Service Units or Facilities. These are not the laboratories where experimental work takes place – although sometimes procedures, such as feeding special diets or administering drugs are carried out there – rather these are the spaces where animals used in experiments are bred and kept, and where they live out the majority of their lives.

2 Named Animal Care and Welfare Officer.

3 Named Training and Competency Officer.

4 During the course of this research both authors were becoming new mothers. This led to some moments of reflection on, and feelings of kinship with, breeding research animals: At one point I watch a mother feeding her litter. I think my own recent pregnancy and breastfeeding has made me particularly sensitive to this. I recall the tug of a baby feeding on the breast and the strangely (probably hormone fuelled) pleasurable sensation. I feel particularly glad of the extra attention Jack gave the pregnant mum. (Research Diary Extract, September 2013) At the same time, knowing the fate of the mouse litter highlighted the very different degrees of control and power we and the animals we observed held over our fates; how would I feel if someone removed my son the moment he was weaned?

5 All individual names have been replaced by pseudonyms to protect participant confidentiality. All interview material was recorded, transcribed and quoted with the informed consent of all human participants.

6 While there is no space to dwell on this here, in the course of this research, both of us at times were both engaged by the animals and people we worked with, but also experienced moments of challenge and distress when faced with, for example, watching animals being culled and the effects this had on the people who performed this work.

## References

[bibr1-0263775818807720] Acampora R (2006) Corporeal Compassion. Pittsburgh: University of Pittsburgh Press.

[bibr2-0263775818807720] Adams R (1977) The Plague Dogs. London: Allen Lane.

[bibr3-0263775818807720] Anderson K (1995) Culture and nature at the Adelaide Zoo: At the frontiers of ‘human’ geography. Transactions of the Institute of British Geographers 20: 275–294.

[bibr4-0263775818807720] Atwood M (2009) Oryx and Crake. London: Virago.

[bibr5-0263775818807720] Bastian M Jones O Moore N et al (2017) Participatory Research in More-than-Human Worlds. London: Routledge.

[bibr6-0263775818807720] Bear C (2011) Being Angelica? Exploring individual animal geographies. Area 43: 297–304.

[bibr7-0263775818807720] Bennett J (2010) Vibrant Matter: A Political Ecology of Things. Durham: Duke University Press.

[bibr8-0263775818807720] Birke L Arluke A Michael M (2007) The Sacrifice: How Scientific Experiments Transform Animals and People. Lafayette: Purdue University Press.

[bibr9-0263775818807720] Buller H (2004) Where the wild things are: The shifting iconography of animals in rural space. Journal of Rural Studies 20: 131–141.

[bibr10-0263775818807720] Buller H (2015) Animal geographies II methods. Progress in Human Geography 39(3): 374–384.

[bibr11-0263775818807720] Buller H Roe E (2018) Food and Animal Welfare: Producing and Consuming Valuable Lives. London: Bloomsbury.

[bibr12-0263775818807720] Candea M (2013) Habituating meerkats and redescribing animal behaviour science. Theory, Culture & Society 30(7–8): 105–128.

[bibr13-0263775818807720] Davies G (2012) Caring for the multiple and the multitude: Assembling animal welfare and enabling ethical critique. Environment and Planning D: Society and Space 30: 623–638.

[bibr14-0263775818807720] Davies GF Greenhough BJ Hobson-West P et al (2016) Developing a collaborative agenda for humanities and social scientific research on laboratory animal science and welfare. PLoS ONE 11(7): e0158791. 27428071 10.1371/journal.pone.0158791 PMC4948886

[bibr15-0263775818807720] Despret V (2004) The body we care for: Figures of anthropo-zoo-genesis. Body & Society 10(2–3): 111–134.

[bibr16-0263775818807720] Despret V (2013) Responding bodies and partial affinities in human–animal worlds. Theory, Culture & Society 30(7/8) 51–76.

[bibr17-0263775818807720] Doughman E (2015) Just how effective is the mouse grimace scale? Available at: www.alnmag.com/news/2015/10/just-how-effective-mouse-grimace-scale (accessed 27 December 2017).

[bibr18-0263775818807720] Druglitro T (2016) Care and tinkering in the animal house In: Bjorkdahl K Druglitro T (eds) Animal Housing and Human-Animal Relations: Politics, Practices and Infrastructures. Abingdon: Routledge, pp. 151–166.

[bibr19-0263775818807720] Druglitro T (2017) ‘Skilled Care’ and the making of good science. *Science, Technology, & Human Values*.

[bibr20-0263775818807720] Fowler KJ (2014) We are all Completely Beside Ourselves. London: Serpent’s Tail.

[bibr21-0263775818807720] Friese C (2013) Realizing potential in translational medicine: The uncanny emergence of care as science. Current Anthropology 54(S7):129–138.

[bibr22-0263775818807720] Fudge E (2011) Pest friends In: Snæbjörnsdóttir B Wilson M (eds) Uncertainty in the City. Berlin: The Green Box, pp. 50–71.

[bibr23-0263775818807720] Geiger M Horkova AJ (2015) Animal performativity: Exploring the lives of donkeys in Botswana. Environment and Planning D: Society and Space 33(6): 1098–1117.

[bibr24-0263775818807720] Gillespie K (2016) Witnessing animal others: Bearing witness, grief, and the political function of emotion. Hypatia 31(3): 572–588.

[bibr25-0263775818807720] Greenhough B Roe E (2010) From ethical principles to response-able practice. Environment and Planning D: Society and Space 28(1): 43–45.

[bibr26-0263775818807720] Greenhough B Roe E (2011) Ethics, space and somatic sensibilities: Comparing relationships between scientific researchers and their human and animal experimental subjects. Environment and Planning D 29: 47–66.

[bibr27-0263775818807720] Greenhough B Roe E (2017) Exploring the role of animal technologists in implementing the 3Rs: An ethnographic investigation of the UK University Sector, Science, Technology and Human Values 43: 694–722. 10.1177/0162243917718066 PMC6027776 30008494

[bibr28-0263775818807720] Greenhough B (2012) Where species meet and mingle: Endemic human-virus relations, embodied communication and more-than-human agency at the Common Cold Unit 1946–90. Cultural Geographies 19(3): 281–301.

[bibr29-0263775818807720] Giraud E Hollin G (2016) Care, laboratory beagles and affective utopia. Theory, Culture & Society 33(4): 27–49.

[bibr30-0263775818807720] Haraway D (1997) Modest-Witness@second-Millenium.femaleMan-Meets OncoMouse. Oxford: Routledge.

[bibr31-0263775818807720] Haraway D (2008) When Species Meet (Posthumanities). Minneapolis: University of Minnesota Press.

[bibr32-0263775818807720] Haraway D (2016) Staying with the Trouble: Making Kin in the Chthulucene. Durham: Duke University Press.

[bibr33-0263775818807720] Hayward E (2010) Fingeryeyes: Impressions of cup corals. Cultural Anthropology 25(4): 577–599.

[bibr34-0263775818807720] Hinchliffe S (2015) More than one world, more than one health: Re-configuring interspecies health. Social Science & Medicine 129: 28–35. 25022470 10.1016/j.socscimed.2014.07.007

[bibr35-0263775818807720] Hodgetts T Lorimer J (2014) Methodologies for animals’ geographies: Cultures, communication and genomics. Cultural Geographies 22(2): 285–295.

[bibr36-0263775818807720] Holmberg T (2011) Mortal love: Care practices in animal experimentation. Feminist Theory 12(2): 147–163.

[bibr37-0263775818807720] Home Office (2016) *Annual statistics of scientific procedures on living animals Great Britain 2016* Available at: https://www.gov.uk/government/statistics/statistics-of-scientific-procedures-on-living-animals-great-britain-2016 (accessed 17 October 2018)

[bibr38-0263775818807720] Johnston E (2015) Of lobsters, laboratories, and war: Animal studies and the temporality of more-than-human encounters. Environment and Planning D: Society and Space 33: 296–313.

[bibr39-0263775818807720] Kirk RG (2016) Care in the cage: Materialising moral economies of animal care in the biomedical sciences In: Bjorkdahl K Driglitro T (eds) Animal Housing and Human-Animal Relations: Politics, Practices and Infrastructures. Routledge: Abingdon, pp. 167–184.

[bibr40-0263775818807720] Kirksey SE Helmreich S (2010) The emergence of multispecies ethnography. Cultural Anthropology 25(4): 545–576.

[bibr42-0263775818807720] Langford DJ Bailey AL Chanda ML et al (2010) Coding of facial expressions of pain in the laboratory mouse. Nature Methods 7(6): 447–449. 20453868 10.1038/nmeth.1455

[bibr43-0263775818807720] Lorimer H (2006a) Herding memories of humans and animals. Environment and Planning D: Society and Space 24: 497–518.

[bibr44-0263775818807720] Lorimer J (2006b) What about the nematodes? Taxonomic partialities in the scope of UK biodiversity conservation. Social & Cultural Geography 7(4): 539–558.

[bibr45-0263775818807720] Lorimer J (2007) Nonhuman charisma. Environment and Planning D 25(5): 911–932.

[bibr46-0263775818807720] Lorimer J (2008) Counting corncrakes: The affective science of the UK corncrake census. Social Studies of Science 38(3): 377–405.

[bibr47-0263775818807720] Lulka D (2008) The paradoxical nature of growth in the US bison industry. Journal of Cultural Geography 25: 31–56.

[bibr48-0263775818807720] Mol A (2008) The Logic of Care: Health and the Problem of Patient Choice. Abingdon: Routledge.

[bibr49-0263775818807720] Puig de la Bellacasa M (2011) Matters of care in technoscience: Assembling neglected things. Social Studies of Science 41 (1): 85–106. 21553641 10.1177/0306312710380301

[bibr50-0263775818807720] Puig de la Bellacasa M (2012) ‘Nothing comes without its world’: Thinking with care The Sociological Review 60(2): 197–216.

[bibr51-0263775818807720] Svendsen MN Koch L (2013) Potentializing the research piglet in experimental neonatal research. Current Anthropology 54(S7): S118–S128.

[bibr52-0263775818807720] Tsing A (2015) The Mushroom at the End of the World: On the Possibility of Life Amidst Capitalist Ruins. Princeton: Princeton University Press.

[bibr53-0263775818807720] Van Dooren T (2014) Flightways: Life and Loss at the Edge of Extinction. New York: Columbia University Press.

[bibr54-0263775818807720] Van Dooren T Kirksey E Munster U (2016) Multispecies studies: Cultivating arts of attentiveness. Environmental Humanities 8(1): 1–23.

[bibr55-0263775818807720] Whatmore S Thorne L (1998) Wild(er)ness: Reconfiguring geographies of wildlife. Transactions of the Institute of British Geographers 23(4): 435–454.

